# Leaderless consensus decision-making determines cooperative transport direction in weaver ants

**DOI:** 10.1098/rspb.2023.2367

**Published:** 2024-08-14

**Authors:** Daniele Carlesso, Madelyne Stewardson, Donald James McLean, Geoffrey P. F. Mazué, Simon Garnier, Ofer Feinerman, Chris R. Reid

**Affiliations:** ^1^ School of Natural Sciences, Macquarie University, New South Wales 2109, Australia; ^2^ Department of Biological Sciences, New Jersey Institute of Technology, Newark, NJ, USA; ^3^ Department of Physics of Complex Systems, Weizmann Institute of Science, Rehovot, Israel

**Keywords:** collective behaviour, leadership, swarm intelligence, wisdom-of-the-crowd, self-organization, emergent behaviour

## Abstract

Animal groups need to achieve and maintain consensus to minimize conflict among individuals and prevent group fragmentation. An excellent example of a consensus challenge is cooperative transport, where multiple individuals cooperate to move a large item together. This behaviour, regularly displayed by ants and humans only, requires individuals to agree on which direction to move in. Unlike humans, ants cannot use verbal communication but most likely rely on private information and/or mechanical forces sensed through the carried item to coordinate their behaviour. Here, we investigated how groups of weaver ants achieve consensus during cooperative transport using a tethered-object protocol, where ants had to transport a prey item that was tethered in place with a thin string. This protocol allows the decoupling of the movement of informed ants from that of uninformed individuals. We showed that weaver ants pool together the opinions of all group members to increase their navigational accuracy. We confirmed this result using a symmetry-breaking task, in which we challenged ants with navigating an open-ended corridor. Weaver ants are the first reported ant species to use a ‘wisdom-of-the-crowd’ strategy for cooperative transport, demonstrating that consensus mechanisms may differ according to the ecology of each species.

## 1. Introduction

Consensus is central to the lives of social animals. Whether they be politicians voting laws or honeybees selecting a nesting site, groups must be able to agree on one option out of mutually exclusive ones [1]. Effective decision-making strategies solve conflicts between individuals and, in turn, prevent group fragmentation. Studying information processing and integration within groups is thus central to understanding the dynamics that allow groups to maintain cohesion.

Strategies for achieving consensus may range from following a small minority of influential individuals (‘leaders’), to pooling together the opinions of all group members. Evidence for leadership behaviour has been reported for several animal groups [2], including humans [3,4], canid packs [5,6] and bee swarms [7,8]. Leaders may have greater influence on the group’s decisions because they move faster [8,9] or are larger [10,11] than their companions, or because they exhibit strong directional preferences [12]. The same exists in human groups when individuals are unable to verbally communicate with each other [3,13]. A different but equally widespread strategy is the ‘wisdom-of-the-crowd’ [14–16]. It states that, as long as each group member makes an independent assessment of the available options, the accuracy of the group will increase with its size. Indeed, the inaccuracy of each individual’s assessment is largely cancelled out by the summing of many opinions. This principle has received extensive support in both human and animal behaviour literature [14,15,17–21]. These two strategies are best interpreted as the two extremes along a continuum of consensus decision-making mechanisms. Intermediate strategies that combine the two exist, and may be highly effective depending on the context in which animals make decisions.

An excellent example of consensus decision-making is cooperative transport, where two or more individuals carry an item together to a destination. Mostly found in humans and ants [22–25], cooperative transport requires consensus on which direction to move in. Unlike humans, ants cannot verbally communicate their intentions to the rest of the group. Instead, they rely on chemical signals and mechanical forces sensed through the carried items [23,25,26]. Furthermore, decisions made by individual ants are based only on locally available information [23,24]. The decentralized nature of cooperative transport makes it extremely resilient to individual failure, adaptable to environmental conditions and scalable with group size.

Currently reported in more than 40 ant genera [22,27], cooperative transport allows colonies to greatly expand the range of prey items that workers can retrieve. In some species, such as *Lasius neoniger* and *Pheidole oxyops*, cooperatively transported items can constitute up to 85% of the total food mass arriving at the colony [28,29]. Cooperative transport is found in ant species that occupy vastly different ecological contexts with distinct environmental challenges. This makes cooperative transport an excellent model system for studying how consensus decision-making may evolve under different environmental pressures. Most commonly, ants encircle the prey item from all directions so that some ants face the desired direction of motion while others face the opposite direction [24]. Carriers must coordinate their efforts to achieve efficient transport and avoid getting stuck in a useless tug-of-war. Several studies have investigated the dynamics underlying cooperative transport in ants [24,30–35], but how the decisions of each worker are integrated within the group has been seldom explored so far. A notable exception is that of the longhorn crazy ant *Paratrechina longicornis*, which relies on leadership behaviour for navigating during cooperative transport [25,26].

**Figure 1.**
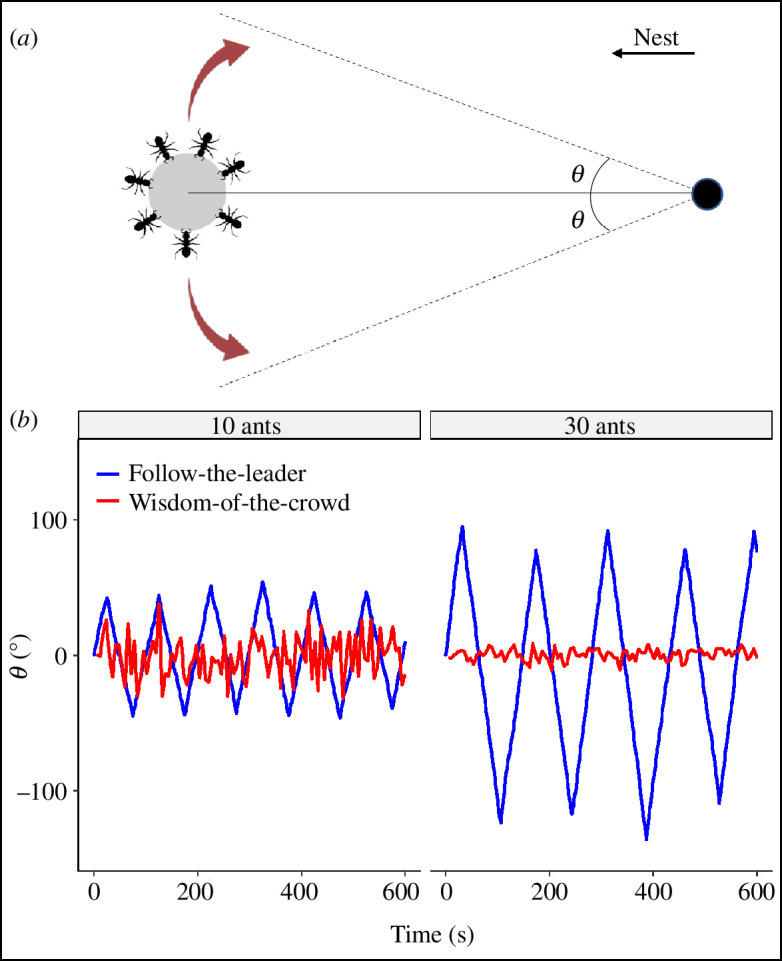
Schematic (*a*) and predictions (*b*) for the tethered-object protocol. (*a*) Shows a simplified diagram of the tethered-object protocol. The item is connected to a string (solid line) to a fixed point in the arena, and the angular deviations θ from the average direction of motion are measured. (*b*) The plot shows predictions of the angular deviations produced by small (left) and large (right) groups using a ‘wisdom-of-the-crowd’ (red) or a ‘follow-the-leader’ (blue) strategy. Predictions were generated by simulating trajectories in R, based on data from [34].

Comparative approaches for studying decision-making in animal groups are rare. Gelblum *et al.* [34] proposed a tethered-object approach to investigate the behavioural mechanisms underlying cooperative transport. Here, the presence of an obstacle is simulated by tethering a prey item to a fixed point of the arena using a thin string, and the magnitude of the angular deviations that groups produce around their average direction of movement is measured (figure 1*a*). This decouples the motion produced by leaders and followers, revealing the consensus strategy used by the group. Simulations of the ‘follow-the-leader’ and ‘wisdom-of-the-crowd’ strategies in a statistical physics model revealed starkly different group movement patterns [25,34] (figure 1*b*). Groups using the ‘follow-the-leader’ strategies produce regular periodic oscillations around their average direction of motion, the amplitude of which increases with group size. This is because only a small minority of informed ants (leaders) actively steers the item in the desired direction, whereas most carriers simply try to keep it going in the current direction, which is in this case tangential. This leads to the emergence of periodic oscillations [34]. Larger group sizes lead to increased behavioural inertia [36], which manifests as deterministic oscillations with larger magnitude. The 'wisdom-of-the-crowd’ strategy leads to random non-periodic fluctuations that decrease in magnitude as group size increases. This is because all carriers possess some knowledge of where they are heading, although this may be noisy, and the group’s direction of motion emerges by the averaging of their estimates of the correct heading. The group’s accuracy increases with the number of its members, because the error contained in each ant’s estimate is cancelled out by the averaging of all estimates during navigation. The tethered-object protocol allows us to position ant species along a one-dimensional spectrum of consensus mechanisms, where oscillatory motion indicates leadership behaviour while random non-periodic fluctuations indicate information pooling.

This simple set-up represents an excellent comparative approach for studying consensus decision-making across ant species. In the original study [34], *P. longicornis* transport groups produced nearly deterministic oscillations that increased in magnitude with group size, providing support for the ‘follow-the-leader’ consensus model (figure 1*b*). The same oscillatory motion allows *P. longicornis* groups to collectively solve navigational challenges without the need for individual workers to be aware of obstacles along the way [25,37]. For instance, when faced with a wall perpendicular to their path, *P. longicornis* groups oscillate between the two ends of the obstacle with little to no change in movement speed or stopping rate, until eventually exiting the wall from one side [25,26,32,34]. The tethered-object protocol has also been tested on *Pheidole pallidula* ants, a species that occupies a similar ecological niche to *P. longicornis* but is phylogenetically distant from it [25]. *Pheidole pallidula* groups generated large-scale oscillations around their average heading, indicating that they use a ‘follow-the-leader’ strategy and thus a convergent evolution towards leadership behaviour in the two species.

Here, we used the tethered-object approach to study cooperative transport in the weaver ant *Oecophylla smaragdina*. Weaver ants are a polydomous and arboreal species that inhabits tropical Australasian regions [38,39], where they are dominant [40,41]. They display an impressive range of cooperative behaviours [41–43], and can integrate visual, magnetic and chemical cues for navigation [44]. *Oecophylla* ants are opportunistic predators that feed on arthropods and small vertebrates [41,45], and they have been observed transporting very large prey items moved by hundreds of cooperating foragers [45]. Given the wide range of prey sizes that they can collect, together with their polydomous nesting habit and excellent navigational skills, weaver ants are an attractive system to study cooperative transport. The aim of our study is twofold. First, we investigate the consensus strategy that weaver ants use during cooperative transport. Second, we want to establish the tethered-object protocol as a comparative tool for studying cooperative transport across ant species [25]. We challenged ant groups of different size with transporting tethered items (grasshoppers), measuring their angular deviations from their average direction of motion. If the ants use a ‘follow-the-leader’ consensus model, we will observe periodic oscillations that increase in magnitude with group size. If ants use a ‘wisdom-of-the-crowd’ strategy, we will instead observe random non-periodic fluctuations with magnitude inversely proportional to group size (figure 1*b*). We further predict that the decrease in fluctuation magnitude will be faster than one over the square root of group size, because the standard deviation of the force applied by *N* ants will decrease as *N* increases (electronic supplementary material, §1). We then challenged groups in an untethered, symmetry-breaking task, where they had to negotiate their exit from an open-ended corridor to retrieve an item back to the nest. Since the ‘follow-the-leader’ strategy allows groups to promptly reach directional consensus, we expect these groups to oscillate between the two ends of the corridor until eventually exiting from either side, as observed in *P. longicornis* [25,26,32,34]. These groups show large reversal rates but little change in movement speed and stopping rate while within and outside the corridor. Groups using a ‘wisdom-of-the-crowd’ strategy will instead experience greater conflict in directional preferences within the corridor than outside of it, because the directional estimates of ants will only align once the obstacle has been escaped. We thus expect large changes in the groups’ movement statistics when within and outside of the corridor. Specifically, after exiting the corridor, the groups’ movement speed will increase, while their stopping and reversal rates will decrease.

## 2. Material and methods

### (a) Experimental set-up

Experiments were performed using nine queenright colonies (~2000 workers) of the weaver ant *O. smaragdina* (Fabricius 1775) between December 2021 and December 2022, and May 2024. Colonies were maintained in temperature-controlled rooms at 27 ± 1°C under a 12:12 h photoperiod at Macquarie University (Sydney, Australia), and fed twice a week with 50% (v/v) sugar water and crickets.

The foraging arena consisted of a white plastic box (64 × 40 × 22 cm) with walls coated with talcum powder to prevent the ants escaping [46]. The arena was positioned in a metal enclosure (150 × 83 × 150 cm) covered with white curtains and evenly illuminated from multiple directions to eliminate orientational cues. The floor of the arena was covered with a paper sheet that was replaced at the end of each testing day (tethered-object experiment) or at the end of each trial (corridor experiment) to remove chemical traces left by ants. The floor of the arena was cleaned with 100% ethanol to remove residual chemical traces at the end of each day. Experimental trials were video recorded using a Panasonic GH4 camera in 4K resolution (3840 × 2160 pixel) at 24 or 25 p.

We used canned grasshoppers (Exo Terra^©^ Grasshoppers XL) as prey items in all experiments. We presented ants with grasshoppers weighing 0.3, 1.3 or 3.5 (±10%) g to cause variation in the number of ants carrying the load. Hereafter, we refer to these stimuli as *light*, *medium* and *heavy* items, respectively. *Heavy* items were obtained by inserting standard magnets (1 g each) in the thorax of the grasshoppers. *Light* items were obtained by cutting grasshoppers into smaller pieces. *Medium* items were generally obtained using full grasshoppers. Items were weighed daily. We used fresh grasshoppers every day and maintained them at room temperature (20 ± 1°C) between trials to avoid desiccation.

### (b) Experimental methodology

We starved colonies for 24 h before testing to ensure high foraging motivation. On testing days, colonies were connected to the experimental arena through a plastic tube (ø 22 mm). To incentivize recruitment of ants to the arena, we placed a feeder containing 50% sucrose solution and a pinned grasshopper on top of a clean A4 paper sheet in the centre of the arena. Ants were allowed to explore the new environment for at least 30 min before testing. Trials started when ants successfully formed foraging trails to the food sources. Colonies that failed to form foraging trails within 1 h were deemed unmotivated and replaced. We removed the A4 paper sheet along with the food sources from the arena before starting experiments.

#### (i) Tethered-object experiment

We tethered a grasshopper to the floor of the arena using a thin 10 cm long string. The grasshopper was initially positioned near its tethering point on top of a clean A4 paper sheet, which ensured no chemical contamination between trials. Ants had to transport the item for 10 cm before fully extending the string. We video recorded trials for 10 min after ants fully extended the string. At the end of each trial, we removed the A4 paper sheet and the tethered item from the arena. All ants connected to the item were then returned untagged to the colony. Given that experiments were performed using colonies consisting of ~2000 workers, it is very unlikely that transport parties were composed of the same foragers over successive trials, minimizing the likelihood of learning or familiarity effects (electronic supplementary material, figures S15 and S16). Colonies were tested a maximum of six times a day with 30 min rest between trials. All colonies were presented with each item weight (light, medium, heavy) at least once in pseudo-random order across trials. We performed 11 trials for *light* items, and 12 trials for *medium* and *heavy* items (total *n* = 35).

#### (ii) Corridor experiment

We placed a LEGO^©^ corridor (30 × 5 cm) 25 cm from the nest entry, perpendicular to the ants’ direction of entrance, and positioned a grasshopper at the centre of the corridor in a vertical orientation to prevent directional biases (electronic supplementary material, figure S8). We then allowed ants to enter the arena and started video recording. Trials lasted until the ants successfully transported the item to the nest entrance, at which point a new trial was set up. Colonies were not allowed to consume the cricket. Colonies that failed to move the item within 45 min of the start of the experiment were deemed unmotivated and replaced. We tested colonies a maximum of five times a day with 30 min rest between trials. The order of presentation of items was pseudo-randomized across trials, ensuring that every colony was presented with each item weight (light, medium, heavy) at least once. We performed 10 trials for each experimental condition (total *n* = 30).

In a follow-up experiment, we challenged groups (*n* = 6) to retrieve a prey item (cricket, 0.3 g) from a blind-ended corridor with the same dimensions and set-up as detailed above. This ensured that all ants entered the corridor from the same side, controlling for effects introduced by the obstacle itself and not related to the degree of intragroup conflict during navigation.

### (c) Data extraction and analysis

The position of the item was extracted for each frame using a custom computer vision script coded in R [47] (v. 4.3.1) using the *Rvision* [48] and *trackR* packages [49]. For the tethered-object experiment, the script returned the (x, y) coordinates of the item’s centroid and the angle of its main axis relative to the vertical axis of the image. For the corridor experiment, the script also returned the traffic rate and directionality of ants at both ends of the corridor. All data were scaled using a ruler in frame. The position and number of carriers attached to the item were manually noted every 720 frames. Ants were considered as carriers if their mandibles overlapped with the item without moving for the 10 frames preceding and following each frame of interest, allowing us to distinguish between ants carrying the item and ants that were transiently sensing it with their mouthparts. This also allowed us to compare the ants’ distribution around items across conditions (electronic supplementary material, figure S10). Data manipulation was performed in R using the packages *tidyverse* [50], *data.table* [51], *circular* [52] and *trajr* [53].

In the tethered-object experiment, we quantified the fluctuations produced by the group around its average direction of motion for 10 min. We calculated the average direction of motion within each trial and assigned it a vector of 0 degrees. This allowed us to compare fluctuations across trials. The angle of deviation from the average direction of motion was then calculated for each frame of the video recordings.

In the corridor experiment, we analysed the trajectory of the item for each trial using the *trajr* package in R. Trajectories within the corridor included the movement of groups from the moment they moved 2 cm until the moment they exited the obstacle. This ensured that our analyses excluded the ‘uncoordinated’ phase of transport, which is typically dominated by an initial build-up of carriers and their exploratory behaviour towards the prey item, and ensured that our data only included the ‘coordinated’ phase of transport where intragroup conflict in the desired heading can be observed [23,24]. Trajectories outside of the corridor started after groups travelled 3 cm from the end of the obstacle and finished when they arrived within 3 cm of the nest entrance. This ensured that these trajectories did not include any residual conflict carried over by the group from the obstacle navigation phase. These exclusion criteria ensured that any difference in the trajectories’ statistics within and outside of the corridor would be due to the degree of intragroup conflict in the ants’ directional opinions rather than other concurrent factors—such as recruitment strategies or insufficient number of carriers. We smoothed all trajectories before analysis to reduce tracking noise, and calculated the speed, moving speed, number of stops, proportion of path backtracked and straightness for each trajectory, each defined below.

We considered groups to have stopped when their speed was extremely slow (below 0.1662 mm s^−1^). This threshold was arbitrarily calculated as the 0.15 quantile of the speed distribution across all trajectories within the corridor. The speed and moving speed of the load were calculated as the average speed between each pair of trajectory points, measured in mm s^−1^. Moving speed refers to the speed of the group when removing all segments of the trajectory in which ants were stopped (speed < 0.1662 mm s^−1^). The number of stops was measured as the number of times that the speed of the group fell below 0.1662 mm s^−1^. We standardized the likelihood of groups to stop by dividing the number of stops by the duration of the trajectory (stops s^−1^). The proportion of path backtracked was calculated as the ratio between the total distance that ants travelled in the direction opposite to the final direction of motion (i.e. if ants escaped the corridor from the right exit, the total distance backtracked is the distance that ants travelled towards the left exit) and the total length of the trajectory. Path straightness was calculated by dividing the total distance travelled by the group by the shortest distance between the initial and final points of the trajectory. Thus, path straightness can only assume numbers between 0 and 1 where 1 indicates a perfectly straight trajectory. Traffic rate and directionality were measured in ants s^−1^ at both ends of the corridor.

We measured the coordination among carriers using the efficiency index “ R ” [23]. R represents the rate of delivery as a flow rate per worker, measured in grams-metre per second per worker. We calculated this index as:


$$\text{R}=\frac{mS}{N}$$

where m is the item weight, S is the average speed of groups and N is the average group size of ants carrying the item. We used average speed rather than directional velocity so as to compare the coordination efforts of groups within and outside of the corridor.

### (d) Statistical analyses

Statistical analyses were performed in R using the *lme4* [54], *glmmTMB* [55], *lmerTest* [56] and *ggeffects* [57] packages. We used generalized linear mixed-effects models (GLMMs) to compare measurements across experimental conditions. Unless otherwise specified, all GLMMs were fit using the *glmmTMB* package. Model diagnostics were performed using the *DHARMa* package [58]. Since we wanted to compare the performance of groups across conditions, rather than test the absolute effect of item weight, we included item weight as a categorical predictor in all models. We performed *post hoc* pairwise comparisons between item weights (*light–medium; light–heavy; medium–heavy*) and their interaction with position in the arena (within or outside of corridor) using the *emmeans* package [59].

#### (i) Tethered-object experiment

We tested the effect of item weight on carrier group size using a GLMM with Gamma distribution and square-root link. The model included the average number of ants as a dependent variable, item weight as a fixed effect and colony ID as a random effect.

The magnitude of the fluctuations produced by groups was calculated as the range (maximum − minimum) of angle deviations from their average direction of motion. We compared fluctuations across conditions using a GLMM with Gaussian distribution. We log-transformed the response variable to ensure residual normality and reduce heteroscedasticity. The model included the log-transformed magnitude of fluctuations as a response variable, item weight as a fixed effect and colony ID as a random effect.

We calculated the average load per ant by dividing item weight by the number of carriers. We tested the effect of item weight on the load per ant using a GLMM with Gamma distribution and log link, which included load per ant as a response variable, item weight as a fixed effect and colony ID as a random effect. Item weight was also set as a dispersion parameter to reduce variance heterogeneity.

#### (ii) Corridor experiment

We used GLMMs to test whether the average speed, stopping rate, distance backtracked and path straightness shown by ant groups were influenced by their position in the arena (within or outside of the corridor), by item weight and/or by the load transported by each ant. Measurements were averaged over the time in which groups were within or outside the corridor. All GLMMs included item weight and position in the arena as categorical predictors, the interaction between these two variables, load per ant as a continuous predictor and trial ID as a random effect. Average group speed was tested using a GLMM with Gamma distribution and log link. Average moving speed was tested using a GLMM with Gamma distribution and log link, which also included item weight as a dispersion parameter to reduce overdispersion. Stopping rates were compared using a GLMM with Tweedie distribution and log link. This distribution is appropriate for modelling non-negative values that include zeros [60]. Since both distance backtracked and path straightness of groups are proportions in the closed interval (0, 1), these measurements were compared across conditions using an ordered beta regression in *glmmTMB*.

We tested the effects of item weight and position in the arena on group size and load per ant using GLMMs with Gamma distribution and log link. Models included item weight and position in the arena as fixed effects, the interaction between the two, and trial ID as a random effect. We used a GLMM with Gaussian distribution to test whether the efficiency of groups (“ R ”) was influenced by item weight, position in the arena or the interaction between the two. Trial ID was included as a random effect. We performed pairwise comparisons using Watson’s two-sample test for homogeneity to test whether the distribution of ants around the item’s centroid depended on the groups’ position in the arena—both generally and for each item weight.

## 3. Results

### (a) Tethered-object experiment

**Figure 2.**
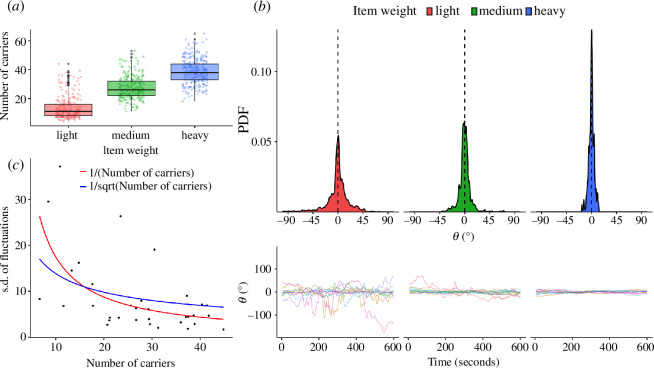
Results of the tethered-object experiment. (*a*) Boxplot showing median, interquartile range, minimum and maximum values of the number of ants engaged in cooperative transport as a function of item weight. Black dots indicate outliers, while coloured dots show individual measurements. (*b*) Upper panel: density plot showing the magnitude of fluctuations produced by ant groups as a function of item weight. Black dashed line indicates the average direction of motion of groups. Lower panel: angle deviations shown by ant groups transporting light, medium and heavy items as function of time; 0 indicates the average direction of motion of groups. (*c*) Scatter plot showing the relationship between the standard deviation (s.d.) of fluctuations and number of carriers. Each dot represents one experimental trial. Red and blue lines represent fitted models showing that fluctuations decrease as one over the number of carriers (red), and thus faster than one over the square root of the number of carriers (blue).

We verified that manipulating item weight caused a change in the number of ants engaged in cooperative transport (figure 2*a*). As expected, we found that carrier group size increases with item weight (χ^2^ = 159.54, *p* < 0.0001). Pairwise comparisons between item weights confirmed significant differences between each condition (*light–medium: t* = −8.499, *p* < 0.0001; *light–heavy: t* = −11.469, *p* < 0.0001; *medium–heavy: t* = −3.609, *p* = 0.009).

We analysed the trajectory produced by ant groups in each trial using spectral analysis and found no evidence of regular periodicity (figure 2*b*, electronic supplementary material, §2). The magnitude of fluctuations decreased with item weight and, thus, group size (figure 2*b*) (χ^2^ = 33.696, *p* < 0.0001). Groups transporting *light* items produced larger fluctuations compared with groups transporting *medium* (*t* = 3.326, *p* = 0.0065) and *heavy* items (*t* = 5.787, *p* < 0.0001). Accordingly, groups transporting *medium* items showed smaller fluctuations compared with groups transporting *heavy* items (*t* = 2.499, *p* = 0.047).

We calculated the rate at which fluctuations decrease as a function of group size. For each trial, we extracted the standard deviation of the fluctuations produced by ants and averaged the number of ants transporting the item. We found that the magnitude of fluctuations decreases as one over the number of carriers (figure 2*c*). This result is consistent with the notion of ‘wisdom-of-the-crowd’, since the noise decays faster than the upperbound of one over the square root of the number of carriers $\left(1/\sqrt{\left(N\right)}\right)$ that this strategy sets. We found a positive relationship between item weight and load per ant (χ^2^ = 269.3, *p* < 0.0001) (electronic supplementary material, figure S1). Since the observed decrease in magnitude of fluctuations may also be caused by differences in load *per capita*, we extracted the fluctuations produced by groups when carrying different item weights but similar load *per capita* (electronic supplementary material, figure S2). This revealed that groups transporting lighter items produced on average larger fluctuations, even when the load per ant is kept constant (electronic supplementary material, figure S3, *,* §3). Overall, these results are consistent with the hypothesis that weaver ants use a ‘wisdom-of-the-crowd’ strategy when cooperatively transporting items.

### (b) Corridor experiment

We extracted the trajectories of ant groups (electronic supplementary material, figure S4) and found that ants exited the corridor from the right side in 21 out of 30 trials. The side of exit, however, always corresponded to the side with the higher rate of incoming traffic.

**Figure 3.**
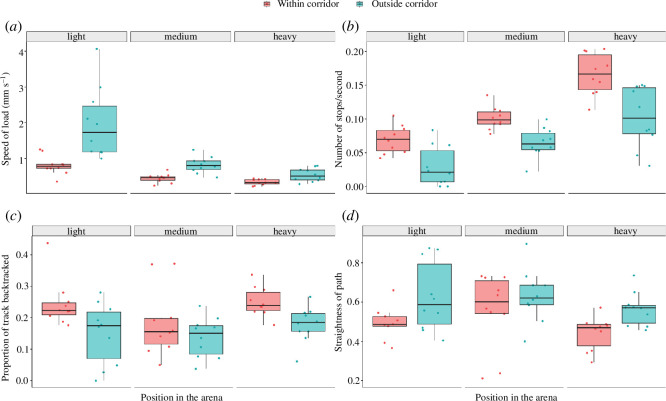
Results of the corridor experiment. Boxplots show median, interquartile range, minimum and maximum values for (*a*) group speed, (*b*) stopping rate, (*c*) proportion of path backtracked and (*d*) path straightness as a function of item weight and position in the arena. Dots show average per replicate.

Group speed was higher for lighter weights (χ^2^ = 9.1115, *p* = 0.0105) and when outside of the corridor (χ^2^ = 70.7911, *p* < 0.0001) (figure 3*a*, electronic supplementary material, table S1). For all item weights, group speed was higher outside of the corridor than within it (*light*: *z* = − 8.414, *p* < 0.0001; *medium*: *z* = −6.276, *p* < 0.0001; *heavy*: *z* = −4.442, *p* < 0.0001). Group speed increased as the load carried by each ant decreased (*z* = −3.904, *p* < 0.0001) (electronic supplementary material, figure S5). A nearly identical pattern in speed was observed when excluding stopping events (electronic supplementary material, figure S7, table S2).

Groups stopped less often when transporting lighter weights (χ^2^ = 13.0533, *p* = 0.0015) and when outside of the corridor (χ^2^ = 14.5396, *p* = 0.0002) (figure 3*b*, electronic supplementary material, table S3). As expected, stopping rates were lower outside of the corridor for all item weights (*light*: *z* = 3.813, *p* = 0.0001; *medium*: *z* = 2.683, *p* = 0.0073; *heavy*: *z* = 3.645, *p* = 0.0003). We found a trend indicating that stopping rates increase with load *per capita* (χ^2^ = 3.7056, *p* = 0.0542).

The proportion of path backtracked by ant groups varied with both item weight (χ^2^ = 6.5569, *p* = 0.0377) and position in the arena (χ^2^ = 7.9104, *p* = 0.0049) (figure 3*c*). Ants backtracked more within the corridor than outside of it when carrying *light* (*z* = 2.813, *p* = 0.0049) or *heavy* items (*z* = 2.119, *p* = 0.0341), but not when carrying *medium* items (*z* = 1.181, *p* = 0.2374). We found no significant difference between groups carrying different item weights either within or outside of the corridor (electronic supplementary material, table S4). Load per ant had no effect (χ^2^ = 0.4418; *p* = 0.5063).

Ant groups travelled straighter paths outside of the corridor than within it (χ^2^ = 9.1171, *p* = 0.0025) (figure 3*d*). Path straightness was higher outside of the corridor for groups transporting *light* items (*z* = −3.019, *p* = 0.0025), but not for groups transporting *medium* (*z* = −1.311, *p* = 0.19) or *heavy* items (*z* = −1.790, *p* = 0.0735). Neither item weight (χ^2^ = 3.8478, *p* = 0.1460) nor load per ant (χ^2^ = 0.4941, *p* = 0.4821) were found to have a significant effect. No significant interaction between position in the arena and item weight was detected (χ^2^ = 1.5673, *p* = 0.4567).

**Figure 4.**
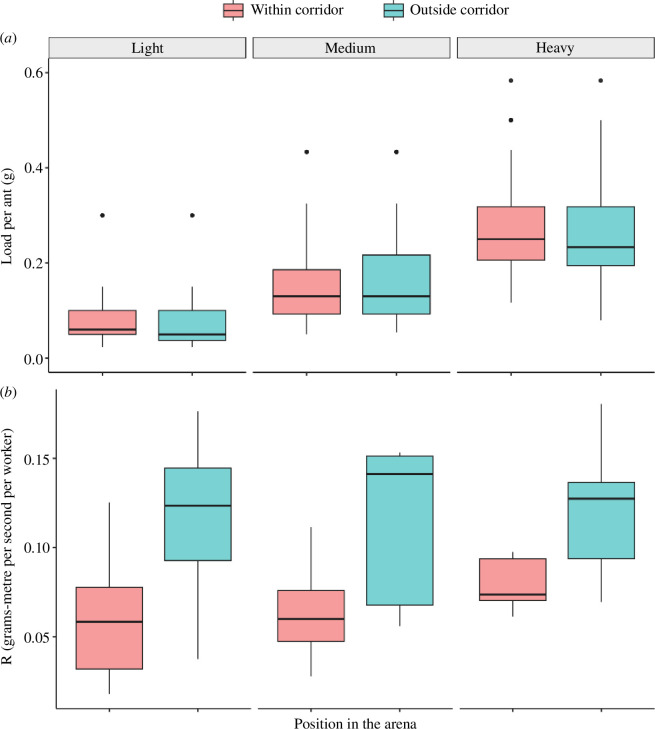
Load *per capita* (*a*) and efficiency (*b*) in the corridor experiment as a function of item weight and position in the arena. Boxplots show the median, interquartile range, maximum and minimum values of the distribution. Black dots show outliers.

Group size increased with item weight (χ^2^ = 40.9891, *p* < 0.0001) but not with the position in the arena (χ^2^ = 3.5988, *p* = 0.0578) (electronic supplementary material, figure S6). No significant interaction between the two predictors was found (χ^2^ = 1.5272, *p* = 0.466). Load *per capita* increased with item weight (χ^2^ = 45.2895, *p* < 0.0001) but did not vary depending on the position in the arena (χ^2^ = 3.6970, *p* = 0.0545) (figure 4*a*). The interaction between these two factors was not significant (χ^2^ = 1.5763, *p* = 0.4547).

Transport efficiency was higher outside the corridor than within it (χ^2^ = 35.1838, *p* < 0.0001) (figure 4*b*). This relationship was consistent across item weights (*light: t* = −5.932, *p* < 0.0001; *medium*: *t* = −5.344, *p* < 0.0001; *heavy*: *t* = −4.216; *p* = 0.0001). Transport efficiency did not vary with item weight (χ^2^ = 1.7833, *p* = 0.410), neither within nor outside of the corridor (electronic supplementary material, table S5). The interaction between position in the arena and item weight was not significant (χ^2^ = 1.5204, *p* = 0.468).

The differences in movement statistics within and outside of the corridor could partially be explained by ants trying to take a direct path to the nest (path integrate) and encountering the obstacle. If this was the case, ants would spend more time in close contact with the wall of the corridor closest to the nest or, alternatively, repeatedly bounce between the two walls. To exclude this hypothesis, we calculated the log-ratio of the distance of the nearest group member to the nest-side wall ( $d_{N}$ ) and the distance of the nearest group member to the opposite wall ( $d_{O}$ ): log ( $\frac{d_{O}}{d_{N}}$ ). If this ratio is centred around 0, then it means that the groups navigate at roughly the same distance from each wall. If the ratio is skewed positively, then the groups navigate closer to the nest-side wall, whereas if the ratio is skewed negatively, the groups navigate closer to the opposite wall. Our data shows that the ratio is generally centred around 0 (mean = 0.30, s.d. = 1.18) (electronic supplementary material, figures S11 and S12), with some groups tending to remain closer to the nest-side wall, others to the opposite wall and some bouncing between the two (electronic supplementary material, figure S13). These results are consistent with widespread observations of thigmotaxis (wall following) in ants, but do not support the alternative hypothesis that path integration explains our results. To further confirm these results, we calculated the ratio between the speed of groups within and outside of the blind-ended corridor. We found that the 95% confidence interval overlapped with a ratio of 1, consistent with the hypothesis that intragroup conflict is reduced when all ants enter from the same direction (electronic supplementary material, figure S14).

## 4. Discussion

Our tethered-object experiment indicated that weaver ants pool together their opinions for navigating during cooperative transport. Groups displayed random non-periodic fluctuations around their average direction of motion, with magnitude inversely proportional to group size (figure 2*b,c*, electronic supplementary material, §2) consistent with the ‘wisdom-of-the-crowd’ strategy [25,34]. This pattern could also be observed when groups transported different item weights while experiencing the same load *per capita* (electronic supplementary material, figures S1 and S2), ruling out alternative explanations such as slower movement or transport difficulty. Our findings contrast with the observations made on *P. longicornis* groups, which produced oscillatory patterns that originated from a ‘follow-the-leader’ navigational strategy [25,34], positioning weaver ants on the opposite end of the consensus continuum from *P. longicornis*.

The ‘wisdom-of-the-crowd’ strategy requires that all ants are roughly informed about the nest position and pull in their preferred direction, without taking into account the forces produced by the other carriers [15]. If ants are truly independent from each other, we expect the standard deviation of the force applied by *N* ants on the item to decrease as one over the square root of N (electronic supplementary material, §1). The timescale of these fluctuations might, however, be too fast to be registered by the empirical angular location of groups, and we consider 1/sqrt(N) to be an upper bound to what the empirical trajectory will show. We expect the magnitude of the fluctuations produced by groups to decrease nonlinearly and faster than one over the square root of group size (1/sqrt(N)). In other words, ant groups should switch direction before exhibiting the full 1/sqrt(N) range of locations. Our findings show that the fluctuations’ magnitude produced by weaver ants decreased as one over *N* (figure 2*c*), consistent with our predictions for the ‘wisdom-of-the-crowd’ consensus model.

Our symmetry-breaking experiment confirmed our results. The presence of a corridor divided the arena in two parts —within and outside of the corridor—varying in the degree of intragroup conflict. We predicted that the higher intragroup conflict would cause groups to be slower, to stop and/or reverse their path more, and to trace more sinuous paths within the corridor than outside of it. All our predictions were confirmed by our results (figure 3*a–d*), and could not be explained by changes in group size (electronic supplementary material, figure S6) or load *per capita* (figure 4*a*). A competing explanation for our results is that groups navigating the corridor tried to take a direct route to the nest (path integrate). The slower movement speed and higher stopping rate could then be explained by ants repeatedly bumping into the wall blocking their path, rather than by the conflict between group members. Our blind-ended corridor experiment failed to discriminate between the two hypotheses. While we observed a reduced difference in transport speed within and outside the corridor (electronic supplementary material, figure S14), groups still moved consistently faster after escaping the obstacle. This could be due to a path integration mechanism, but also to the presence of intragroup conflict within the blind-ended corridor. Ants make decisions based on locally available information, and they may be largely unaware that one side of the obstacle is blocked-off. While we expect the majority of ants to pull towards the direction they came from, this need not be the case and some group members may still pull in the direction of the closed exit. Further experiments should account for this effect, for instance by halving the length of the corridor so that groups immediately encounter a wall if heading towards the blocked exit. To disentangle between the two hypotheses, we analysed the distance between transport groups and the two walls in the open-ended corridor. If ants tried to path integrate, we expect groups to consistently spend the majority of time in close proximity to the nest-side wall. Our observations differ from this prediction, showing that some groups tended to remain closer to the nest-side wall, others to the opposite wall and others bounced between the two (electronic supplementary material, figures S11–S13). These results strongly support the hypothesis that the degree of intragroup conflict rather than path integration mechanisms explains the change in movement statistics observed in our experiments.

The behaviour of *O. smaragdina* and *P. longicornis* groups differ when dealing with obstacles, reflecting the distinct decision-making strategies used by the two species. *Paratrechina longicornis* groups confronted with a wall showed oscillatory movements that are highly reminiscent of those observed in the tethered-object experiment [25,34], which ultimately lead ants to escape the obstacle. Importantly, these groups showed little to no changes in speed or stopping rates while navigating obstacles [25,26,32,35]. *Oecophylla smaragdina* groups, on the other hand, showed no periodic oscillations in our experiments (figure 2*b*, electronic supplementary material, §2). They were efficient in maintaining consensus (figure 4*b*) and rarely retraced their steps or stalled for long periods of time (figure 3*b,c*). In addition, *O. smaragdina* showed dramatic changes in speed and stopping rates after exiting the corridor (figure 3*a,b*). Group speed increased on average by 155, 94 and 56% for light, medium and heavy items, respectively. The stopping rate of groups for light, medium and heavy items was 56, 33 and 41% higher in the corridor than outside of it. These changes cannot be attributed to differences in group size or load *per capita* (figure 4*a*, electronic supplementary material, figure S6), or by ants trying to take a direct route to the nest while navigating the corridor (electronic supplementary material, figures S11–S13). Instead, they emerge from the reduced intragroup conflict once the obstacle is escaped. *Paratrechina longicornis* and *O. smaragdina* groups thus represent excellent examples of the behavioural consequences of using different consensus strategies in the same context.

We cannot explain the slight rightward bias in our corridor experiment. We designed our experiments to minimize directional biases. Experiments were performed within an enclosure fully covered with white curtains, evenly lit from multiple overhead directions. The corridor was positioned centrally in the arena, and the item placed in the middle of the obstacle in a vertical orientation. We minimized chemical contamination by replacing the substrate and the corridor after each replicate. It is unlikely that this bias originated from experience or route learning. The bias was evident in the first trials of each day, where the right side was chosen in 8 out of 11 replicates by ants which had never experienced the arena before. In addition, the time required to navigate the obstacle varied between trials and did not improve in consecutive tests (electronic supplementary material, figure S16). In all cases, however, the exit side chosen by ants corresponded to the side of the corridor with the highest traffic rate. The bias may originate from navigational strategies of ants. Side biases are common in animals [61–65]. Consistent directional turning and outline tracing has been found in other arboreal ants [66], and it may be a conserved strategy for navigation. A small bias in one direction, combined with the positive feedback of pheromone trails, may aid ants achieving consensus by ensuring asymmetrical traffic around obstacles.

A question that arises naturally from our study is why weaver ants use a ‘wisdom-of-the-crowd’ rather than a ‘follow-the-leader’ strategy. While we cannot provide a definitive answer to this question, this may arise from differences in the information available to *O. smaragdina* and *P. longicornis* workers while engaged in cooperative transport. *Paratrechina longicornis* workers lose their bearing soon after joining a transport group, preventing them from navigating [25,26]. Newly attached ants provide groups with updated bearings, transferring environmental information into the system. Weaver ants are exceptional navigators, thanks to their ability to integrate visual, magnetic and olfactory cues [44]. We occasionally observed carriers tapping on the ground with their antennae (Daniele Carlesso, personal observation, 2023), suggesting that they may be able to access pheromone trail information during transport, removing the need for temporary leaders to guide the group. Weaver ants also possess large eyes and rely heavily on visual information for navigation [44,67]. They use celestial cues to derive compass information, but can also use artificial light, distal landmarks or magnetic cues when necessary. Given their remarkable navigational skills, we hypothesize that weaver ants engaged in cooperative transport possess (at least some) knowledge about the route leading back to their nest.

The consensus strategies used by the two species align with the ecological pressures of their natural habitat. *Paratrechina longicornis* ants are opportunistic predators and scavengers that primarily collect proteins from live and dead insects [68,69]. They dominate ecologically, thanks to their exceptional navigational skills and short-range recruitment, outcompeting other species by quickly exploiting food resources [68–72]. The primary goal of cooperative transport in this species is to retrieve prey items as quickly as possible to avoid interspecific competition. The ‘follow-the-leader’ strategy allows them to accomplish this task efficiently, as demonstrated by their ability to navigate obstacles and disordered environments with minimal speed loss [32,35,37]. Conversely, weaver ants are highly territorial and aggressive predators that prey on live arthropods [38,73]. They are opportunistic scavengers, and can cooperatively transport large prey such as small birds [45]. The primary goal of cooperative transport may thus not be rapid movement, as they may prefer to attack approaching competitors rather than escaping them [74]. The ‘wisdom-of-the-crowd’ mechanism may be more robust for transporting live items, as prey body movements may prevent carriers from sensing the direction in which other ants are pulling. Being able to withstand and actively generate forces more than 100 times their own body weight [75,76], *O. smaragdina* is much stronger than *P. longicornis* and among the strongest ants in the world. This may favour slower solutions that rely on brute force rather than faster strategies requiring high coordination among individuals. Future studies on cooperative transport in other species should investigate the links between consensus mechanisms and ecological pressures. For instance, *P. pallidula* ants generate an oscillatory motion when tested in a tethered-object task—indicating a ‘follow-the-leader’ strategy [25]—and share similar ecological pressures to *P. longicornis* [77,78].

One aim of our study was to establish the tethered-object approach as a comparative tool for investigating cooperative transport across ant species [25]. Cooperative transport has been reported in more than 40 ant genera [22,27], but quantitative studies are limited to only a handful of species [24,25,29,30,79]. Even when considering the available research, results are difficult to compare because of differences in methodology. The tethered-object protocol offers an easy-to-run and cost-effective solution to this issue. This protocol simulates the presence of an obstacle, allowing observations of the movement of ant groups over long periods of time [34]. This set-up only requires a prey item, a thin string, an anchor point and a camera. Results can be quickly obtained by tracking the groups’ movement and estimating the number of workers engaged in transport, without the need for advanced individual-level tracking of ants. This approach has proven effective for studying cooperative transport in *P. longicornis* and *P. pallidula* [25], and now in *O. smaragdina*. The tethered-object protocol is limited in that it only describes a linear space of consensus models, delimited at either end by the ‘wisdom-of-the-crowd’ and ‘follow-the-leader’ strategies. Its results are useful to determine which strategy ants are closer to, but not for identifying possible intermediate strategies and their consequences. Given its simplicity and affordability, we believe that this protocol is a valid comparative tool to characterize the consensus strategies of ants during cooperative transport, as well as an excellent candidate for citizen science and educational events [80].

Here, we showed that weaver ants pool together their opinions for achieving navigational consensus during cooperative transport. This allows them to efficiently move along their route and escape obstacles without getting stuck in lengthy deadlocks. The ‘wisdom-of-the-crowd’ strategy has been reported in several animal species [14,81,82], humans [14,83] and even bacteria [84]. Our findings add to the previous literature demonstrating the effectiveness of this strategy in maintaining consensus during collective navigation tasks. A wide survey of cooperative transport is now needed to reveal the relative spread of consensus strategies among ant species and the links with their ecology.

## Data Availability

All code and datasets used for data analyses are available at Zenodo [85]. Supplementary material is available online [86].

## References

[1] Conradt L , Roper TJ . 2005 Consensus decision making in animals. Trends Ecol. Evol. **20** , 449–456. (10.1016/j.tree.2005.05.008) 16701416

[2] Strandburg-Peshkin A , Papageorgiou D , Crofoot MC , Farine DR . 2018 Inferring influence and leadership in moving animal groups. Phil. Trans. R. Soc. B **373** , 20170006. (10.1098/rstb.2017.0006) 29581391 PMC5882976

[3] Dyer JR , Johansson A , Helbing D , Couzin ID , Krause J . 2009 Leadership, consensus decision making and collective behaviour in humans. Phil. Trans. R. Soc. B **364** , 781–789. (10.1098/rstb.2008.0233) 19073481 PMC2689712

[4] Faria JJ , Dyer JRG , Tosh CR , Krause J . 2010 Leadership and social information use in human crowds. Anim. Behav. **79** , 895–901. (10.1016/j.anbehav.2009.12.039)

[5] Peterson RO , Jacobs AK , Drummer TD , Mech LD , Smith DW . 2002 Leadership behavior in relation to dominance and reproductive status in gray wolves, Canis lupus. Can. J. Zool. **80** , 1405–1412. (10.1139/z02-124)

[6] Bonanni R , Cafazzo S , Valsecchi P , Natoli E . 2010 Effect of affiliative and agonistic relationships on leadership behaviour in free-ranging dogs. Anim. Behav. **79** , 981–991. (10.1016/j.anbehav.2010.02.021)

[7] Greggers U , Schöning C , Degen J , Menzel R . 2013 Scouts behave as streakers in honeybee swarms. Naturwissenschaften **100** , 805–809. (10.1007/s00114-013-1077-7) 23812604

[8] Schultz KM , Passino KM , Seeley TD . 2008 The mechanism of flight guidance in honeybee swarms: subtle guides or streaker bees? J. Exp. Biol. **211** , 3287–3295. (10.1242/jeb.018994) 18840663

[9] Pettit B , Ákos Z , Vicsek T , Biro D . 2015 Speed determines leadership and leadership determines learning during pigeon flocking. Curr. Biol. **25** , 3132–3137. (10.1016/j.cub.2015.10.044) 26628007

[10] Reebs SG . 2001 Influence of body size on leadership in shoals of golden shiners, Notemigonus crysoleucas. Behaviour **138** , 797–809. (10.1163/156853901753172656)

[11] Franks NR , Sendova-Franks AB , Anderson C . 2001 Division of labour within teams of new world and old world army ants. Anim. Behav. **62** , 635–642. (10.1006/anbe.2001.1794)

[12] Couzin ID , Krause J , Franks NR , Levin SA . 2005 Effective leadership and decision-making in animal groups on the move. Nature **433** , 513–516. (10.1038/nature03236) 15690039

[13] Dyer JRG , Ioannou CC , Morrell LJ , Croft DP , Couzin ID , Waters DA , Krause J . 2008 Consensus decision making in human crowds. Anim. Behav. **75** , 461–470. (10.1016/j.anbehav.2007.05.010)

[14] Krause J , Ruxton GD , Krause S . 2010 Swarm intelligence in animals and humans. Trends Ecol. Evol. **25** , 28–34. (10.1016/j.tree.2009.06.016) 19735961

[15] Simons AM . 2004 Many wrongs: the advantage of group navigation. Trends Ecol. Evol. **19** , 453–455. (10.1016/j.tree.2004.07.001) 16701304

[16] Galton F . 1907 Vox populi. Nature **75** , 450–451. (10.1038/075450a0)

[17] Couzin ID , Ioannou CC , Demirel G , Gross T , Torney CJ , Hartnett A , Conradt L , Levin SA , Leonard NE . 2011 Uninformed individuals promote democratic consensus in animal groups. Science **334** , 1578–1580. (10.1126/science.1210280) 22174256

[18] Woolley AW , Chabris CF , Pentland A , Hashmi N , Malone TW . 2010 Evidence for a collective intelligence factor in the performance of human groups. Science **330** , 686–688. (10.1126/science.1193147) 20929725

[19] Codling EA , Pitchford JW , Simpson SD . 2007 Group navigation and the “many-wrongs principle” in models of animal movement. Ecology **88** , 1864–1870. (10.1890/06-0854.1) 17645033

[20] Berman S , Lindsey Q , Sakar MS , Kumar V , Pratt SC . 2011 Experimental study and modeling of group retrieval in ants as an approach to collective transport in swarm robotic systems. Proc. IEEE **99** , 1470–1481. (10.1109/JPROC.2011.2111450)

[21] Dell’Ariccia G , Dell’Omo G , Wolfer DP , Lipp HP . 2008 Flock flying improves pigeons’ homing: GPS track analysis of individual flyers versus small groups. Anim. Behav. **76** , 1165–1172. (10.1016/j.anbehav.2008.05.022)

[22] Moffett MW . 2010 Adventures among ants: a global safari with a cast of trillions, 1st edn. Berkeley and Los Angeles, CA: University of California Press. (10.1525/9780520945418)

[23] McCreery HF , Breed MD . 2014 Cooperative transport in ants: a review of proximate mechanisms. Insectes Soc. **61** , 99–110. (10.1007/s00040-013-0333-3)

[24] Czaczkes TJ , Ratnieks FLW . 2013 Cooperative transport in ants (Hymenoptera: Formicidae) and elsewhere. Myrmecol. News **18** , 1–11.

[25] Feinerman O , Pinkoviezky I , Gelblum A , Fonio E , Gov NS . 2018 The physics of cooperative transport in groups of ants. Nat. Phys. **14** , 683–693. (10.1038/s41567-018-0107-y)

[26] Gelblum A , Pinkoviezky I , Fonio E , Ghosh A , Gov N , Feinerman O . 2015 Ant groups optimally amplify the effect of transiently informed individuals. Nat. Commun. **6** , 1–9. (10.1038/ncomms8729) PMC4525283 26218613

[27] Hölldobler B , Wilson EO . 1990 The ants. Berlin, Germany: Harvard University Press. (10.1007/978-3-662-10306-7)

[28] Traniello JFA . 1983 Social organization and foraging success in Lasius neoniger (Hymenoptera: Formicidae): behavioral and ecological aspects of recruitment communication. Oecologia **59** , 94–100. (10.1007/BF00388080) 25024155

[29] Czaczkes TJ , Nouvellet P , Ratnieks FLW . 2011 Cooperative food transport in the Neotropical ant, Pheidole oxyops. Insectes Soc. **58** , 153–161. (10.1007/s00040-010-0130-1)

[30] Buffin A , Pratt SC . 2016 Cooperative transport by the ant Novomessor cockerelli. Insectes Soc. **63** , 429–438. (10.1007/s00040-016-0486-y) PMC6177163 30300423

[31] Buffin A , Sasaki T , Pratt SC . 2018 Scaling of speed with group size in cooperative transport by the ant Novomessor cockerelli. PLoS One **13** , e0205400. (10.1371/journal.pone.0205400) 30300423 PMC6177163

[32] McCreery HF , Dix ZA , Breed MD , Nagpal R . 2016 Collective strategy for obstacle navigation during cooperative transport by ants. J. Exp. Biol. **219** , 3366–3375. (10.1242/jeb.143818) 27807216

[33] McCreery HF . 2017 A comparative approach to cooperative transport in ants: individual persistence correlates with group coordination. Insectes Soc. **64** , 535–547. (10.1007/s00040-017-0575-6)

[34] Gelblum A , Pinkoviezky I , Fonio E , Gov NS , Feinerman O . 2016 Emergent oscillations assist obstacle negotiation during ant cooperative transport. Proc. Natl Acad. Sci. USA **113** , 14615–14620. (10.1073/pnas.1611509113) 27930304 PMC5187715

[35] Ron JE , Pinkoviezky I , Fonio E , Feinerman O , Gov NS . 2018 Bi-stability in cooperative transport by ants in the presence of obstacles. PLoS Comput. Biol. **14** , e1006068. (10.1371/journal.pcbi.1006068) 29746457 PMC5944914

[36] Attanasi A *et al* . 2014 Information transfer and behavioural inertia in starling flocks. Nat. Phys. **10** , 691–696. (10.1038/nphys3035) PMC4173114 25264452

[37] Gelblum A , Fonio E , Rodeh Y , Korman A , Feinerman O . 2020 Ant collective cognition allows for efficient navigation through disordered environments. eLife **9** , e55195. (10.7554/eLife.55195) 32393436 PMC7332297

[38] Hölldobler BK , Wilson EO . 1977 Weaver ants. Sci. Am. **237** , 146–155. (10.1038/scientificamerican1277-146) 841318

[39] Wetterer JK . 2017 Geographic distribution of the weaver ant Oecophylla smaragdina. Asian Myrmecol. **9** , e009004. (10.20362/am.009004)

[40] Blüthgen N , Stork NE . 2007 Ant mosaics in a tropical rainforest in Australia and elsewhere: a critical review. Austral Ecol. **32** , 93–104. (10.1111/j.1442-9993.2007.01744.x)

[41] Holldobler B . 1983 Territorial behavior in the green tree ant (Oecophylla smaragdina). Biotropica **15** , 241–250. (10.2307/2387648)

[42] Carlesso D , McNab JM , Lustri CJ , Garnier S , Reid CR . 2023 A simple mechanism for collective decision-making in the absence of payoff information. Proc. Natl Acad. Sci. USA **120** , e2216217120. (10.1073/pnas.2216217120) 37428910 PMC10629567

[43] Bochynek T , Robson SK . 2014 Physical and biological determinants of collective behavioural dynamics in complex systems: pulling chain formation in the nest-weaving ant Oecophylla smaragdina. PLoS One **9** , e95112. (10.1371/journal.pone.0095112) 24759886 PMC3997362

[44] Jander R , Jander U . 1998 The light and magnetic compass of the weaver ant, Oecophylla smaragdina (Hymenoptera: Formicidae). Ethology **104** , 743–758. (10.1111/j.1439-0310.1998.tb00108.x)

[45] Wojtusiak J , Godzińska EJ , Dejean A . 1995 Capture and retrieval of very large prey by workers of the African weaver ant, Oecophylla longinoda (Latreille 1802). Trop. Zool. **8** , 309–318. (10.1080/03946975.1995.10539287)

[46] Ning D , Yang F , Xiao Q , Ran H , Xu Y . 2019 A simple and efficient method for preventing ant escape (hymenoptera: formicidae). Myrmecol. News. **29** , 57–65. (10.25849/myrmecol.news_029:057)

[47] R Core Team . 2023 R: A Language and Environment for Statistical Computing. R Foundation for Statistical Computing, Vienna, Austria. See https://www.r-project.org/.

[48] Garnier S , Muschelli J . 2024 Rvision - a computer vision library for R. R package version 0.8.0. See https://github.com/swarm-lab/Rvision.

[49] Garnier S . 2022 trackR - multi-object tracking with R. R package version 0.5.3. See https://github.com/swarm-lab/trackR.

[50] Wickham H *et al* . 2019 Welcome to the Tidyverse. J. Open Source Softw. **4** , 1686. (10.21105/joss.01686)

[51] Barret T , Dowle M , Arun S , Gorecki J , Chirico M , Hocking T . 2024 Data.table: extension of `data.frame`. R package version 1.15.4.

[52] Agostinelli C , Lund U . 2023 R package 'circular': Circular Statistics (version 0.5-0). See https://CRAN.R-project.org/package=circular.

[53] McLean DJ , Skowron Volponi MA . 2018 trajr: an R package for characterisation of animal trajectories. Ethology **124** , 440–448. (10.1111/eth.12739)

[54] Bates D , Mächler M , Bolker B , Walker S . 2015 Fitting linear mixed-effects models using lme4. J. Stat. Softw. **67** , 1–48. (10.18637/jss.v067.i01)

[55] Brooks ME , Kristensen K , Benthem KJ , Magnusson A , Berg CW , Nielsen A *et al* . 2017 glmmTMB balances speed and flexibility among packages for zero-inflated generalized linear mixed modeling. R J. **9** , 378–400. (10.32614/RJ-2017-066)

[56] Kuznetsova A , Brockhoff PB , Christensen RHB . 2017 lmerTest package: tests in linear mixed effects models. J. Stat. Softw. **82** , 1–26. (10.18637/jss.v082.i13)

[57] Lüdecke D . 2018 ggeffects: tidy data frames of marginal effects from regression models. J. Open Source Softw. **3** , 772. (10.21105/joss.00772)

[58] Hartig Florian . 2022 DHARMa: residual diagnostics for hierarchical (multi-level / mixed) regression models. R package version 0.4.6.

[59] Lenth Russel V . 2024 emmeans: estimated marginal means, aka least-squares means. R package version 1.10.2

[60] Gilchrist R , Drinkwater D . 2000 The use of the Tweedie distribution in statistical modelling. In: Bethlehem, J.G., van der Heijden, P.G.M. (eds) COMPSTAT. Heidelberg, Germany: Physica. (10.1007/978-3-642-57678-2_39)

[61] Hunt ER , O’Shea-Wheller T , Albery GF , Bridger TH , Gumn M , Franks NR . 2014 Ants show a leftward turning bias when exploring unknown nest sites. Biol. Lett. **10** , 20140945. (10.1098/rsbl.2014.0945) 25540159 PMC4298197

[62] Frasnelli E . 2013 Brain and behavioral lateralization in invertebrates. Front. Psychol. **4** , 939. (10.3389/fpsyg.2013.00939) 24376433 PMC3859130

[63] Cooper R , Nudo N , González JM , Vinson SB , Liang H . 2011 Side-dominance of Periplaneta americana persists through antenna amputation. J. Insect Behav. **24** , 175–185. (10.1007/s10905-010-9246-4)

[64] Kight SL , Steelman L , Coffey G , Lucente J , Castillo M . 2008 Evidence of population-level lateralized behaviour in giant water bugs, Belostoma flumineum say (Heteroptera: Belostomatidae): T-maze turning is left biased. Behav. Processes **79** , 66–69. (10.1016/j.beproc.2008.04.001) 18490112

[65] Girling R , Hassall M , Turner J . 2007 Do turning biases by the 7-spot ladybird, Coccinella septempunctata, increase their foraging efficiency? Behaviour **144** , 143–163. (10.1163/156853907779947337)

[66] Jander R . 1990 Arboreal search in ants: search on branches (Hymenoptera: Formicidae). J. Insect Behav. **3** , 515–527. (10.1007/BF01052015)

[67] Ogawa Y , Jones L , Ryan LA , Robson SKA , Hart NS , Narendra A . 2023 Physiological properties of the visual system in the green weaver ant, Oecophylla smaragdina. J. Comp. Physiol. A **209** , 489–498. (10.1007/s00359-023-01629-7) PMC10354137 37055584

[68] Kenne M , Mony R , Tindo M , Kamaha Njaleu LC , Orivel J , Dejean A . 2005 The predatory behaviour of a tramp ant species in its native range. C. R. Biol. **328** , 1025–1030. (10.1016/j.crvi.2005.09.001) 16286091

[69] Witte V , Attygalle AB , Meinwald J . 2007 Complex chemical communication in the crazy ant Paratrechina longicornis latreille (Hymenoptera: Formicidae). Chemoecology **17** , 57–62. (10.1007/s00049-006-0364-6)

[70] Wetterer JK *et al* . 1999 Ecological dominance by Paratrechina longicornis (Hymenoptera: Formicidae), an invasive tramp ant, in biosphere 2. Fla. Entomol. **82** , 381–388. (10.2307/3496865)

[71] Latty T , Beekman M . 2013 Keeping track of changes: the performance of ant colonies in dynamic environments. Anim. Behav. **85** , 637–643. (10.1016/j.anbehav.2012.12.027)

[72] Czaczkes TJ , Vollet-Neto A , Ratnieks FLW . 2013 Prey escorting behavior and possible convergent evolution of foraging recruitment mechanisms in an invasive ant. Behav. Ecol. **24** , 1177–1184. (10.1093/beheco/art046)

[73] Hölldobler B , Wilson EO . 1983 The evolution of communal nest-weaving in ants: steps that may have led to a complicated form of cooperation in weaver ants can be inferred from less advanced behavior in other species. Am. Sci. **71** , 490–499.

[74] Rastogi N . 2000 Prey concealment and spatiotemporal patrolling behaviour of the Indian tree ant Oecophylla smaragdina (Fabricius). Insectes Soc. **47** , 92–93. (10.1007/s000400050014)

[75] Federle W , Rohrseitz K , Hölldobler B . 2000 Attachment forces of ants measured with a centrifuge: better “wax-runners” have a poorer attachment to a smooth surface. J. Exp. Biol. **203** , 505–512. (10.1242/jeb.203.3.505) 10637179

[76] Stewardson M . 2023 Many ants make light work; quantifying force output of weaver ant teams. Sydney, Australia: Macquarie University. (10.25949/22672081)

[77] Detrain C , Deneubourg JL . 1997 Scavenging by Pheidole pallidulaa key for understanding decision-making systems in ants. Anim. Behav. **53** , 537–547. (10.1006/anbe.1996.0305)

[78] Detrain C . 1990 Field study on foraging by the polymorphic ant species, Pheidole pallidula. Insectes Soc. **37** , 315–332. (10.1007/BF02225995)

[79] Wang C , Chen X , Strecker R , Henderson G , Wen X , Hooper-Bùi LM . 2016 Individual and cooperative food transport of the red imported fire ant (Hymenoptera: Formicidae): laboratory observations. J. Insect Behav. **29** , 99–107. (10.1007/s10905-016-9546-4)

[80] Silvertown J . 2009 A new dawn for citizen science. Trends Ecol. Evol. **24** , 467–471. (10.1016/j.tree.2009.03.017) 19586682

[81] Ward AJW , Sumpter DJT , Couzin ID , Hart PJB , Krause J . 2008 Quorum decision-making facilitates information transfer in fish shoals. Proc. Natl Acad. Sci. USA **105** , 6948–6953. (10.1073/pnas.0710344105) 18474860 PMC2383955

[82] Sasaki T , Granovskiy B , Mann RP , Sumpter DJT , Pratt SC . 2013 Ant colonies outperform individuals when a sensory discrimination task is difficult but not when it is easy. Proc. Natl Acad. Sci. USA **110** , 13769–13773. (10.1073/pnas.1304917110) 23898161 PMC3752206

[83] Kurvers RHJM , Herzog SM , Hertwig R , Krause J , Wolf M . 2021 Pooling decisions decreases variation in response bias and accuracy. iScience **24** , 102740. (10.1016/j.isci.2021.102740) 34278254 PMC8267549

[84] Moreno-Gámez S , Hochberg ME , van Doorn GS . 2023 Quorum sensing as a mechanism to harness the wisdom of the crowds. Nat. Commun. **14** , 3415. (10.1038/s41467-023-37950-7) 37296108 PMC10256802

[85] Carlesso D , Stewardson M , Donald JM , Garnier S , Feinerman O , Reid RC . 2024 Code and datasets for: Leaderless consensus decision-making determines cooperative transport direction in weaver ants. Zenodo. (10.5281/zenodo.12738277) PMC11323088 39140325

[86] Carlesso D , Stewardson M , Garnier S , Feinerman O , Reid CR . 2024 Supplementary material from: Leaderless consensus decision-making determines cooperative transport direction in weaver ants. Figshare. (10.6084/m9.figshare.c.7399611) PMC11323088 39140325

